# A clinical-grade, lightweight multimodal foundation model for closed-loop telemedicine

**DOI:** 10.1093/bib/bbaf631.038

**Published:** 2025-12-12

**Authors:** Xuan Gao, Sicheng Zheng

**Affiliations:** Key Laboratory of Artificial Organs and Computational Medicine in Zhejiang Province, Shulan International Medical College, Zhejiang Shuren University, Hangzhou, 310015, P. R. China; University of Hong Kong, Hong Kong; Key Laboratory of Artificial Organs and Computational Medicine in Zhejiang Province, Shulan International Medical College, Zhejiang Shuren University, Hangzhou, 310015, P. R. China

## Abstract

**Aims:**

This study aims to overcome the limitation of integrated AI in telemedicine by developing MedAssist, an end-to-end intelligent auxiliary system designed to leverage multimodal patient data across the entire care continuum.

**Methods:**

We built MedAssist upon a novel lightweight multimodal medical foundation model, establishing a standardized data platform and a specialized model architecture to fuse textual and visual data.^1^ The model was compressed using knowledge distillation and quantization-aware training to enable rapid deployment while maintaining high accuracy.

**Results:**

Evaluated on a multi-center dataset, the model achieved an AUC of 0.87 in a critical diagnostic task and reduced inference latency by 60%. A pilot clinical validation demonstrated a 40% reduction in triage time and a 92% concordance rate with senior physician diagnoses.

**Conclusion:**

The findings indicate that MedAssist provides a robust and scalable framework for enhancing the efficiency and quality of remote healthcare, representing a significant step towards practical, AI-augmented telemedicine.

**References:**

1. Vorontsov, E., Bozkurt, A., Casson, A. et al. **A foundation model for clinical-grade computational pathology and rare cancers detection.** Nature Medicine 2024; **30**, 2924–2935.

